# Optimal repetition time reduction for single subject event‐related functional magnetic resonance imaging

**DOI:** 10.1002/mrm.27498

**Published:** 2018-09-19

**Authors:** Amy R. McDowell, David W Carmichael

**Affiliations:** ^1^ UCL GOS Institute of Child Health London UK; ^2^ EPSCRC / Wellcome Centre for Medical Engineering Kings College London UK

**Keywords:** autoregressive model, EEG‐fMRI, event related, fMRI, multiband, simultaneous multislice

## Abstract

**Purpose:**

Short TRs are increasingly used for fMRI as fast sequences such as simultaneous multislice excitation become available. These have been associated with apparent sensitivity improvements, although greater temporal autocorrelation at shorter TRs can inflate sensitivity measurements leading to uncertainty regarding the optimal approach.

**Methods:**

In volunteers (*n* = 10), the optimal TR was assessed at the single subject level for event‐related designs (visual stimulation) with 4 frequencies of presentation at 4 TR values (412‐2550 ms). T‐values in the visual cortex localized in each individual were obtained and receiver operating characteristics (ROC) analysis was performed by counting voxels within and outside expected task active regions at different thresholds. This analysis was repeated using 4 different autoregressive (AR) models; SPM AR(1) and SPM AR(fast) which globally estimate autocorrelation, and fMRIstat AR(1) and AR(5) that use a local estimate.

**Results:**

The use of modest multiband factors of 2 or 3 with a reduction in TR to 1000 ± 200 ms had greater sensitivity and specificity as shown by higher T‐values in visual cortex and ROC analysis. At these TRs, the ROC analysis demonstrated that a local AR model fit improved performance while high order AR models were unnecessary.

**Conclusions:**

Modest TR reductions (to 1000 ± 200 ms) optimally improved event‐related fMRI performance independent of design frequency. Autoregressive models with a local as opposed to global fit performed better, while low order autoregressive models were sufficient at the optimal TR.

## 1 INTRODUCTION

Sequences suitable for fMRI with short TRs, such as simultaneous multislice excitation which allows for several slices of MRI data to be obtained at the same time,^1^, ^2^, ^3^, ^4^, ^5^ have become widely available. This makes it possible to maintain volume coverage and TE while reducing TR for gradient EPI (GE‐EPI) that is typically used for fMRI.

Of particular importance in the assessment of improvements in fMRI results is the appropriate control of false positives. Reduced TR sequences with much greater temporal sampling can inflate statistical results because the degrees of freedom of the data can appear much larger than standard TR sequences. However, typical analyses (e.g., based on the general linear model) assume that each data point is independent of the next, (i.e., noise found at 1 time point is uncorrelated with noise at the next time point). This assumption is clearly violated in fMRI data and so autocorrelations are modelled. However, as the TR decreases (TR<<hemodynamic response function) the degree of autocorrelation increases and may become more complex both temporally (e.g., due to physiological noise) and spatially (e.g., due to g‐factor related noise enhancement). It is, therefore, imperative to control for this autocorrelation in the data which will increase as the TR is reduced. A recent study by Sahib et al^6^ found that stable statistics were dependent on the TR and AR model order. However, this study focused on sensitivity by means of t‐values for a single event‐related design based on a single patient with epilepsy and did not evaluate false positives.

In this study, we investigated the effect of TR and event rate on fMRI sensitivity using a visual stimulus paradigm. We sought to establish optimal TRs at the single subject level for event‐related designs with differing frequency of fMRI events by estimating false positive rates and performing an receiver operating characteristics (ROC) curve analysis. In addition, we evaluated the impact of autoregressive models on these results.

## 2 METHODS

### 2.1 Subjects

Ten healthy adult volunteers (6 male, 3 female; average age, 32 years; range, 22‐48 years) participated in this study after providing informed consent.

### 2.2 fMRI paradigm

**Figure 1:**
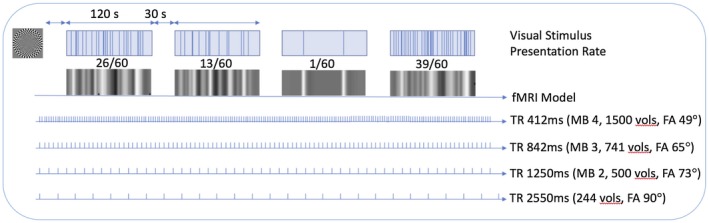
Paradigm design diagram showing checkerboard pattern used and the visual stimulus PR (top row), this is convolved with the canonical hemodynamic response function (second row) to form a model of expected signal changes. Based on the sequence parameters these signal changes are sampled at different rates (bottom 4 rows). TR is the repetition rate in milliseconds, FA is the flip angle, vols is the number of imaging volumes

To simulate varying event rates, a paradigm was used that comprised of rest blocks viewing a fixation cross, and 4 event blocks consisting of viewing the fixation cross interspersed with a checkerboard pattern which was presented with a duration of 300 ms at pseudorandom intervals. Pseudorandom intervals were generated in Matlab using a random number generator with the same seed value for each session. The checkerboard pattern is shown in Figure 1.

The paradigm consisted of a rest block of 30 s, followed alternately by four 120‐s event blocks and four 30‐s rest blocks. Each event block had a different event presentation rate (PR) of 1/60 s, 13/60 s, 26/60 s, 39/60 s presented in the same order of 26/60 s, 13/60 s, 1/60 s, and 39/60 s for each session. The range of event rates was based on epileptic discharge patterns seen in previously scanned epileptic patients,^7^, ^8^ and was an average rate over the event block as the checkerboard was presented at pseudorandom intervals. The checkerboard was presented on an liquid crystal display screen (NNL, Bergen, Norway) situated at the head end of the scanner bore which was viewed by the subject using a mirror attached to the head coil.

### 2.3 Image acquisition

Imaging data was acquired with a 3T scanner and 64‐channel head/neck coil (Siemens Prisma, Erlangen, Germany). Structural T_1_‐weighted images were obtained with an MP‐RAGE sequence (TR 2300 ms, TE 2.74 ms, inversion time 909 ms, voxel size 1 × 1 × 1 mm^3^). Event‐related fMRI data were obtained with GE‐EPI, and a voxel size of 2.5 × 2.5 in‐plane and 2.5 mm^3^ slices with a 0.5‐mm gap, matrix 80 × 80, 40 slices. Four sessions of 10 min with different simultaneous multislice speed up or multiband (MB) factors were used (see Figure 1).

We used a maximum MB = 4 based on previous work that demonstrated increased false positives due to aliasing artefacts at higher MB factors.^9^ The flip angle for each session was calculated as the Ernst angle with a T_1_ of 1000 ms as a reasonable average value between measured white and gray matter T_1_ values.^10^

#### 2.3.1 Localization of the primary visual cortex

To independently define the location of the maximum response in the visual cortex of each volunteer, a 2‐min V1 localizer (TR 2550 ms, TE 27 ms, flip angle 90°, 60 vol) was first acquired with two 40‐s blocks, alternated with 20‐s rest blocks (60 events, checkerboard as above).

### 2.4 Image processing / general linear model analysis

All images were first preprocessed using statistical parametric mapping software (SPMv12,^11^, ^12^ https://www.fil.ion.ucl.ac.uk/spm) for use with both SPM and fMRIstat. Preprocessing included image realignment, which consisted of co‐registration of fMRI time series data from each session to the mean, and then smoothing with a Gaussian filter using the default SPM value (8 mm FWHM). A high‐pass filter with a cutoff frequency of 1/128 Hz was used in all analyses to mitigate low‐frequency signal components.

#### 2.4.1 Localization of the primary visual cortex

The localizer was analyzed first. The visual stimulus was modelled in a standard block design using the default canonical hemodynamic response function and default autoregressive model of SPM AR(1). The global maximum from the positive t‐contrast of the visual stimulus blocks was examined and the spatial location recorded. This location for each individual was used in all subsequent analysis. Any subjects without a global maximum within visual cortex for the localizer were not considered in subsequent analysis.

#### 2.4.2 Variable TR and stimulus frequency analysis

Analysis was performed both in SPM and fMRIstat^13^ (https://www.math.mcgill.ca/keith/fmristat/). In both cases, the 6 realignment parameters were used as effects of no interest and the smoothed realigned images were the input data. Separate GLM models were built for each of the 4 sessions (as the data had differing TRs) with 4 separate conditions (i.e., 1 regressor for each presentation frequency) using the timing of individual checkerboard presentations as an event relate design. These were convolved with the canonical hemodynamic response function using the default model within SPM and fMRIstat.

### 2.5 Autoregressive models

We wished to investigate how t‐values were influenced by different autoregressive models. SPM provides an AR(1) model for standard TR values and AR(fast) for use with shorter TRs. SPMs AR(1) model uses an 1^st^ order expansion (approximately ω = 0.2) that is fitted globally to the covariance. SPMs AR(fast) uses a dictionary of exponential covariance components that are fitted globally to the covariance. We compared these with 2 AR models available within fMRIstat, which allows the order of the AR model to be selected. We chose fMRIstat AR(1) to provide a comparison of consistency with the SPM AR(1), and AR(5) based on Sahib et al.^6^ The second difference is that fMRIstat uses a local (per voxel) fit of AR coefficients and noise (ω) from least square residuals using Yule‐Walker‐equations.^13^ Therefore, the 4 imaging sessions were analyzed with 4 different AR models; (1) SPM AR(1), (2) SPM AR(fast), (3) fMRIstat AR(1), and (4) fMRIstat AR(5). The resulting SPM and fMRIstat maps of all t‐values in all voxels within the brain were then co‐registered by taking the mean of each session and co‐registering it to the mean of the V1 localizer and applying the transformation to the corresponding t‐value maps. The t‐value at each PR, TR, and frequency was determined at the subject specific location of V1 (the global maximum from the separate localizer session).

### 2.6 Statistical analysis

The V1 t‐value at each PR, TR, and AR model was entered into the statistical analysis. The mean and standard deviation of the t‐value across subjects for each TR and presentation frequency combination was calculated.

A second level analysis using a 2‐way repeated measures analysis of variance with factors TR and frequency was used to evaluate their effect on the t‐values using SPSS version 24 (https://www.ibm.com/pk-en/marketplace/spss-statistics). To test the effect of TR reduction with conservative control of potential t‐value inflation at shorter TRs,^6^ the 2‐way analysis of variance compared t‐values obtained with the standard SPM AR1 model for the standard TR value (2550 ms) and t‐values obtained using the fMRIstat AR(5) model at shorter TRs (412, 842, and 1250 ms). Significance for a factor or interaction was taken to be *P* < 0.05. Mauchly's Test of Sphericity was used to test that the variances of the differences between all combinations of related groups were equal. Posthoc paired t‐tests were run to compare t‐values obtained at each TR. Significance was again taken to be *P* < 0.05 after Bonferroni correction.

An analysis of sensitivity and specificity at each TR, frequency and AR model was performed. All subjects/sessions were normalized to MNI space using the ICBM template (European) in SPM. Brain areas were defined as task positive using the group response, evaluated at 2 inclusive levels of significance (p < 0.01 and p < 0.001 uncorrected, >20 contiguous voxels), and applied as a mask to each individual’s map. Individuals activation maps were thresholded at a range of *P*‐values (from *P* < 0.0001‐*P* < 0.5). For each individual, session and threshold the group level mask was applied to voxels within the brain, and a voxel count was performed. True positive voxels were those with t‐values above the threshold within the mask; false negatives were all other voxels within the mask; true negatives were voxels outside the mask below the threshold; false positives were all other voxels within the brain outside the mask. Voxel counts were averaged across subjects and turned into percentage ROC curves plotted and fitted using a 4^th^ order Taylor series in Matlab’s curve fitting toolbox (https://uk.mathworks.com), and areas under the curve (AUC) were then calculated.

## 3 RESULTS

**Figure 2:**
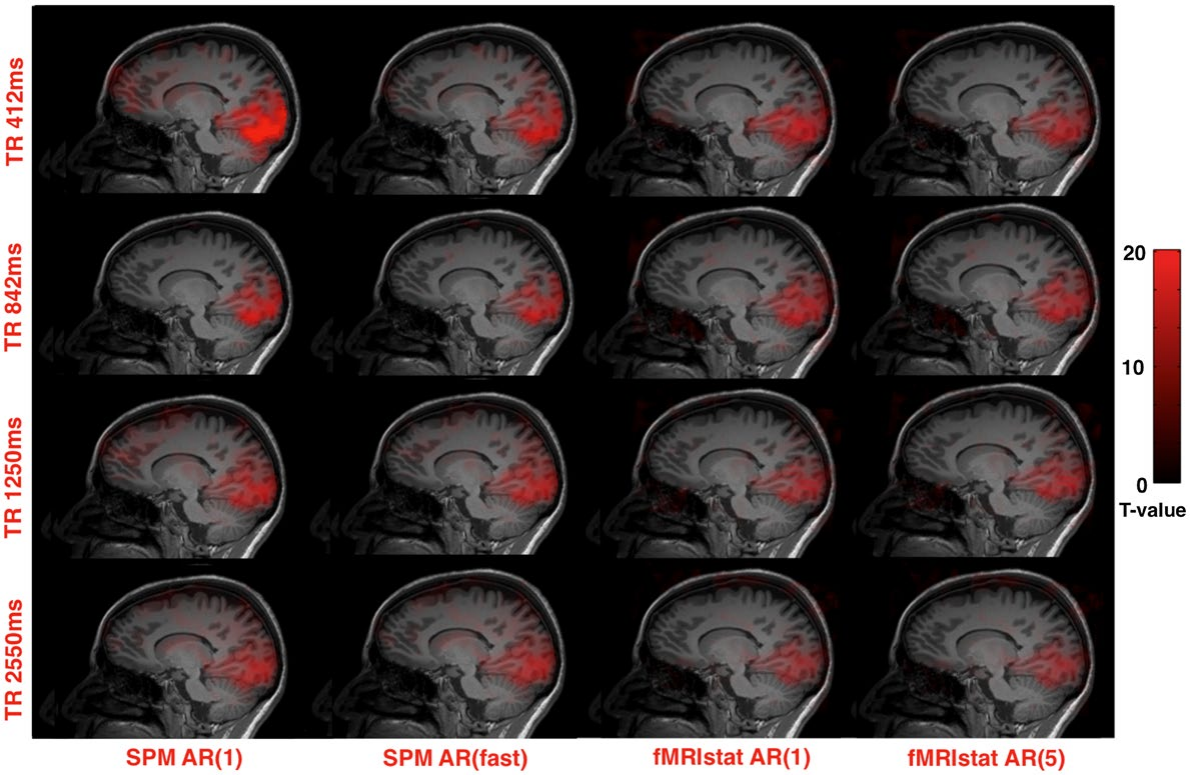
Activation in the visual cortex is illustrated in 1 subject at a threshold of 20 overlaid on their T1 volume. Each image shows the same sagittal slice through the global maximum location. Each panel represents analysis of the same data using different autoregressive models (left to right) and TR (top to bottom)

Of the 10 volunteers, 9/10 showed clear activation to the visual localizer with the global maxima in an anatomical location consistent with primary visual cortex, met the criteria for the study, and so were included in subsequent analysis. Figure 2 shows activation for each TR value and autoregressive model.

**Figure 3:**
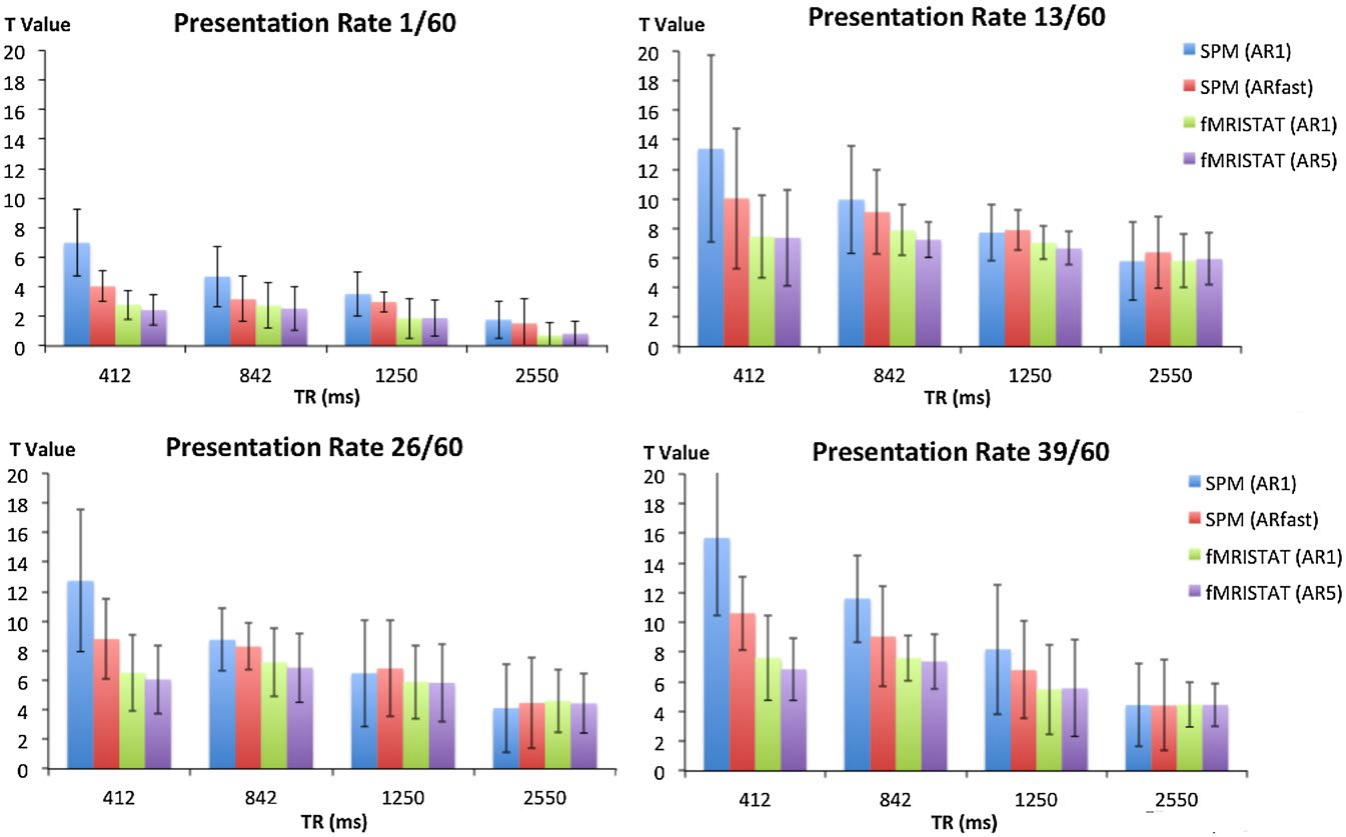
Global maximum t‐values for each TR at PRs of 1/60 (top left), 13/60 (top right), 26/60 (bottom left) and 39/60 (bottom right) for each AR model provided by SPM (AR 1 and fast) and fMRIstat (AR 1 and 5). Error bars are the standard deviation

Figure 3 shows the t‐values in V1 across all subjects for each TR and frequency combination tested with different autoregressive models.

The effect of frequency and TR on the t‐values in V1 was tested with a repeated measured analysis of variance. The assumption of sphericity using Mauchly’s Test was met for TR, χ^2^(2) = 4.28, *P* = 0.515, and frequency of presentation χ^2^(2) = 3.9, *P* = 0.56. The main effects of TR and presentation frequency showed statistically significant differences in t‐value across subjects, F(3, 24) = 6.69, *P* = 0.002, and F(3,24) = 34.497, *P* = <0.001, respectively. There was no interaction between TR and frequency of presentation, F(9,72) = 1.446, *P* = 0.185.

To determine the optimal TR value, posthoc paired t‐tests were performed with the results shown in Table 1. In general, using TRs < 2550 ms increased t‐values. When using a conservative correction for temporal autocorrelation (fMRIstat AR(5)), the t‐value was always increased compared with TR = 2550 ms; an 842‐ms TR increased t‐values by 75% on average. While the 842‐ms TR improved the t‐value compared with 1250 ms, there was no statistical significant increase in t‐value at 412 ms TR compared with either 842 ms or 1250 ms.

**Table 1:** Paired T‐tests comparing t‐values produced using TR 2550 ms and SPM AR1 model against fMRISTAT AR5 model and shorter TR values across all presentation frequencies

TR (ms)	412 ms > 2550 ms	842 ms > 2550 ms	1250 ms > 2550 ms	842 ms > 1250 ms	412 ms > 842 ms	412 ms > 1250 ms
P‐Value	<0.0001	<0.0001	0.0012	0.0007	ns	ns
R^2^	0.3579	0.5515	0.2349	0.2540	0.1654	0.02146
Mean T‐value increase	1.720	2.501	1.424	1.077	−0.7803	0.2963
T‐value increase 95% confidence interval	0.9291 to 2.512	1.672 to 3.329	0.5416 to 2.307	0.4431 to 1.710	−1.382 to −0.1785	−0.3907 to 0.9832
Increase in t‐value (%)	51.3	74.6	42.5	22.5	−15.4	6.2

Choice of autoregressive model made a substantial difference to the measured t‐values as expected. All AR models gave consistent results at the standard TR (2550 ms). The SPM AR(1) model had larger t‐values at shorter TR values. Interestingly, this effect was not solely attributable to the low AR model order, because both the fMRIstat AR(1) and AR(5) models showed a more modest t‐values increase at shorter TR values while the use of the higher order SPM AR(fast) model did not reduce the t‐values to those seen with either fMRIstat model. The AR model coefficients for fMRIstat AR(1) and fMRIstat AR(5) are displayed in Supporting Information Table S1, which is available online.

**Figure 4:**
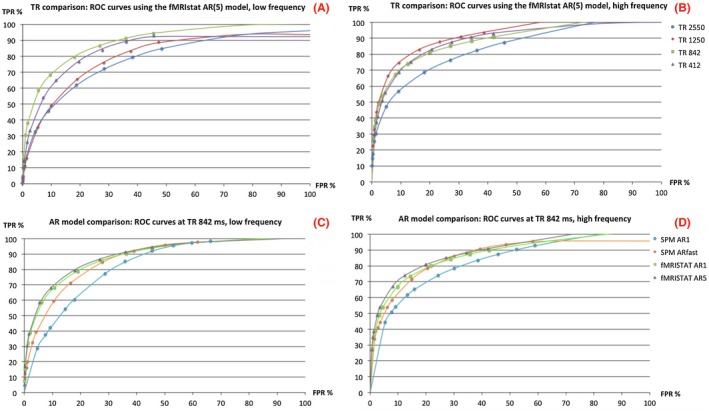
ROC curves for each TR for the lowest (A) and highest (B) rate of presentation obtained with conservative autocorrelation estimates (fMRIstat AR5). ROC curves were also calculated for each autoregressive model at a TR of 842 ms for the lowest (C) and highest (D) rate of presentation. All ROC curves were produced using a group level mask (*P* < 0.001, 20 voxels) to determine true/false positives

ROC curves for each TR with the most conservative model (fMRIstat AR(5)) are shown in Figure 4A,B for the low and high PR, respectively. AUC values (Supporting Information Table S2) were greatest using a TR of 1250 ms at the highest event frequency, and using a TR of 842 ms at the lowest event frequency.

ROC curves are shown for different autoregressive models at a fixed TR (842 ms) at the lowest (Figure 4C) and highest rate of presentation (Figure 4D). The fMRIstat (AR1/5) autoregressive models gave a similar AUC curve at both presentation frequencies. These curves were consistently above the SPM (AR1 and “fast”) autoregressive models indicating improved performance.

These results were generated with a group level mask of task activated brain regions defined using *P* < 0.001. Similar results in terms of optimal TR/ autoregressive model were obtained using *P* < 0.01 (Supporting Information Figure S1).

## 4 DISCUSSION

In this study, we evaluated the improvement in event‐related fMRI sensitivity from using shorter TR, 2D‐SMS sequences. Compared with a standard TR (2550 ms), shorter TRs increased the t‐value for all autoregressive models; with a conservative model (fMRIstat AR(5)) the largest t‐value increase of 75% (Table 1) was found at TR = 842 ms (MB factor 3). ROC curve analysis (Figure 4A,B) demonstrated that a TR of 842‐1250 ms was optimal. At a 5% FPR, there was a clear increase in sensitivity at these TRs. A local fit of autocorrelation was found to be optimal at these TRs with a low order AR model being sufficient.

At shorter TRs image signal is exponentially reduced due to reduced T_1_ recovery within the TR. Therefore, it might be expected that imaging with a shorter TR has a punitive effect on image signal levels that in turn, will reduce fMRI sensitivity, which is consistent with our findings. Our findings demonstrate a more moderate reduction in TR is optimal compared with some previous studies even when a similar autoregressive model was used.^6^ This might be due to differences in the event‐related design, although we found that similar results at different PRs. Slightly different measures of sensitivity were used in the 2 studies (peak t‐values and ROC curves as opposed to mean t‐values). This previous work showed that while a large increase in sensitivity was found using an MB factor of 2 compared with 1, the subsequent increase in performance was modest in comparison (at MB 3‐8), therefore, relatively small experimental differences could result in slight differences in optimal TR. Based on both our ROC curve analysis and previous studies,^9^ higher MB factors/shorter TRs are associated with greater false positives. As expected, the effect of TR on t‐values was found to be highly dependent on the choice of autoregressive model. Performing a local fit of autoregressive coefficients as implemented in fMRIstat provided a much more conservative determination of t‐values irrespective of autoregressive model order. These findings are consistent with Sahib et al,^6^ who also showed that for TRs above 330 ms an AR(1) model with a local fit yielded a stable and conservative statistical result. SPM uses a global autoregressive model fit leading to increased t‐values at shorter TRs with both the AR(1) and AR(fast) variants. It is not possible to determine the underlying “true” t‐value, however, for many clinical and neuroscientific applications a conservative estimate to control for false positive results may be preferable. To overcome this limitation, a ROC curve analysis was performed and it showed that the autoregressive models using a local fit (fMRIstat AR(1 and 5)) performed better.

Our results might indicate a subtle effect of stimulation frequency on the optimal TR (Figure 4A,B where the low frequency stimulus showed slightly higher sensitivity at 1250 ms than 842 ms with the opposite for a high frequency stimulus). However, the interaction between TR and stimulus frequency was not significant.

Our study tested the visual cortex and stimuli within a certain frequency range. Other brain regions, particularly those affected by cardiac noise may have a greater advantage from faster acquisitions. We did not test the effect of noise models on our results. However, improved noise modelling is unlikely to significantly alter our findings, because it is likely to cause a greater decrement in statistical significance when the noise sources are not well sampled (at longer TRs). The impact of subject motion on sensitivity at different TRs remains unclear and shorter TRs could be beneficial in this context although image reconstruction is typically also reliant on calibration data that does not account for subject motion.

There have been several studies investigating the efficacy of SMS sequences for fMRI at the group level; Todd et al^9^ found that higher MB factors showed false activation artefacts due to signal leakage from the simultaneously excited slices, though these were reduced by the use of a Split Slice‐GRAPPA reconstruction, concluding that the MB acceleration factor should be 4 or less for stable behavior which motivated the maximum MB factor used here. Therefore, in addition to providing improved sensitivity, the use of an MB factor of 3 with a TR of ~800 ms should also provide images without strong local noise enhancement and aliasing artefacts as confirmed by our ROC analysis.

Our findings suggest that increasing the speed of data acquisition is beneficial for modest TR reductions (1000 ± 200 ms) and MB factors (2‐3); however, likely owing to the rapid loss of image signal, there are not large advantages for acquisitions with a TR below 800 ms for event‐related fMRI designs at 3T. In the context of increasing artifact levels for greater reduction factors,^9^ this suggests a relatively conservative level of TR reduction is optimal in event‐related applications where single subject results are important. Group studies are less sensitive to improved single subject sensitivity owing to inter‐subject variability.

## Supporting information


**FIGURE S1** ROC curves for each TR for the lowest (A) and highest (B) rate of presentation obtained with conservative autocorrelation estimates (fMRIstat AR5). ROC curves were also calculated for each autoregressive model at a TR of 842 ms for the lowest (C) and highest rate of presentation (D). All ROC curves were produced using a group level mask (*P* < 0.01, 20 voxels) to determine true/false positives
**TABLE S1** Autoregressive model coefficients (AR + ) used by fMRIstat in AR(1) with one coefficient, and AR(5) with five coefficients demonstrating the weighting of the coefficients at higher AR orders by TR
**TABLE S2** Area under the curve values for all models and TR values at the highest and lowest rates of presentation produced using a group level mask (p < 0.001, 20 voxels)
