# Supplementary material for: Optimal repetition time reduction for single subject event‐related functional magnetic resonance imaging
Source: Magn Reson Med. 2018 Sep 19;81(3):1890–7. doi: 10.1002/mrm.27498 (PMC6519282; doi:10.1002/mrm.27498)
Supplement: Supplementary file 1 — FIGURE S1 ROC curves for each TR for the lowest (A) and highest (B) rate of presentation obtained with conservative autocorrelation estimates (fMRIstat AR5). ROC curves were also calculated for each autoregressive model at a TR of 842 ms for the lowest (C) and highest rate of presentation (D). All ROC curves were produced using a group level mask (P < 0.01, 20 voxels) to determine true/false positives TABLE S1 Autoregressive model coefficients (AR + ) used by fMRIstat in AR(1) with one coefficient, and AR(5) with five coefficients demonstrating the weighting of the coefficients at higher AR orders by TR TABLE S2 Area under the curve values for all models and TR values at the highest and lowest rates of presentation produced using a group level mask (p < 0.001, 20 voxels) [file MRM-81-1890-s001.docx]

Supporting Information

Supporting Information Table S1

| Model (coefficient) | TR 412ms | TR 842ms | TR 1250ms | TR 2550ms |
| --- | --- | --- | --- | --- |
| **AR1 (1)** | 0.820 | 0.804 | 0.789 | 0.571 |
| **AR5 (1)** | 0.499 | 0.786 | 0.789 | 0.638 |
| **AR5 (2)** | 0.327 | 0.0059 | 0.113 | -0.188 |
| **AR5 (3)** | 0.131 | 0.053 | 0.081 | 0.074 |
| **AR5 (4)** | -0.0079 | 0.048 | -0.296 | 0.0062 |
| **AR5 (5)** | -0.0055 | -0.184 | 0.103 | -0.0194 |

Supporting Information Table S1: Autoregressive model coefficients (AR + ω) used by fMRIstat in AR(1) with one coefficient, and AR(5) with five coefficients demonstrating the weighting of the coefficients at higher AR orders by TR

Supporting Information Table S2

| Low frequency presentation rate | 412ms | 842ms | 1250ms | 2550ms |
| --- | --- | --- | --- | --- |
| **SPM AR1** | 7505.7 | 8122.3 | 7985.9 | 7629.6 |
| **SPM ARfast** | 8146.4 | 8619.8 | 8171.3 | 7531.6 |
| **fMRIstat AR1** | 8539.5 | 8856.4 | 7864.9 | 7742.2 |
| **fMRIstat AR5** | 8400.6 | 8921.0 | 7966.2 | 7792.4 |
| **High frequency presentation rate** | **412ms** | **842ms** | **1250ms** | **2550ms** |
| **SPM AR1** | 8483.5 | 8294.4 | 8078.5 | 7616.4 |
| **SPM ARfast** | 8172.9 | 8594.5 | 8724.3 | 7751.3 |
| **fMRIstat AR1** | 8574.0 | 8722.5 | 9165.7 | 8420.3 |
| **fMRIstat AR5** | 8922.0 | 8982.8 | 9158.9 | 8382.6 |

Supporting Information Table S2: Area under the curve values for all models and TR values at the highest and lowest rates of presentation

Supporting Information Figure S1


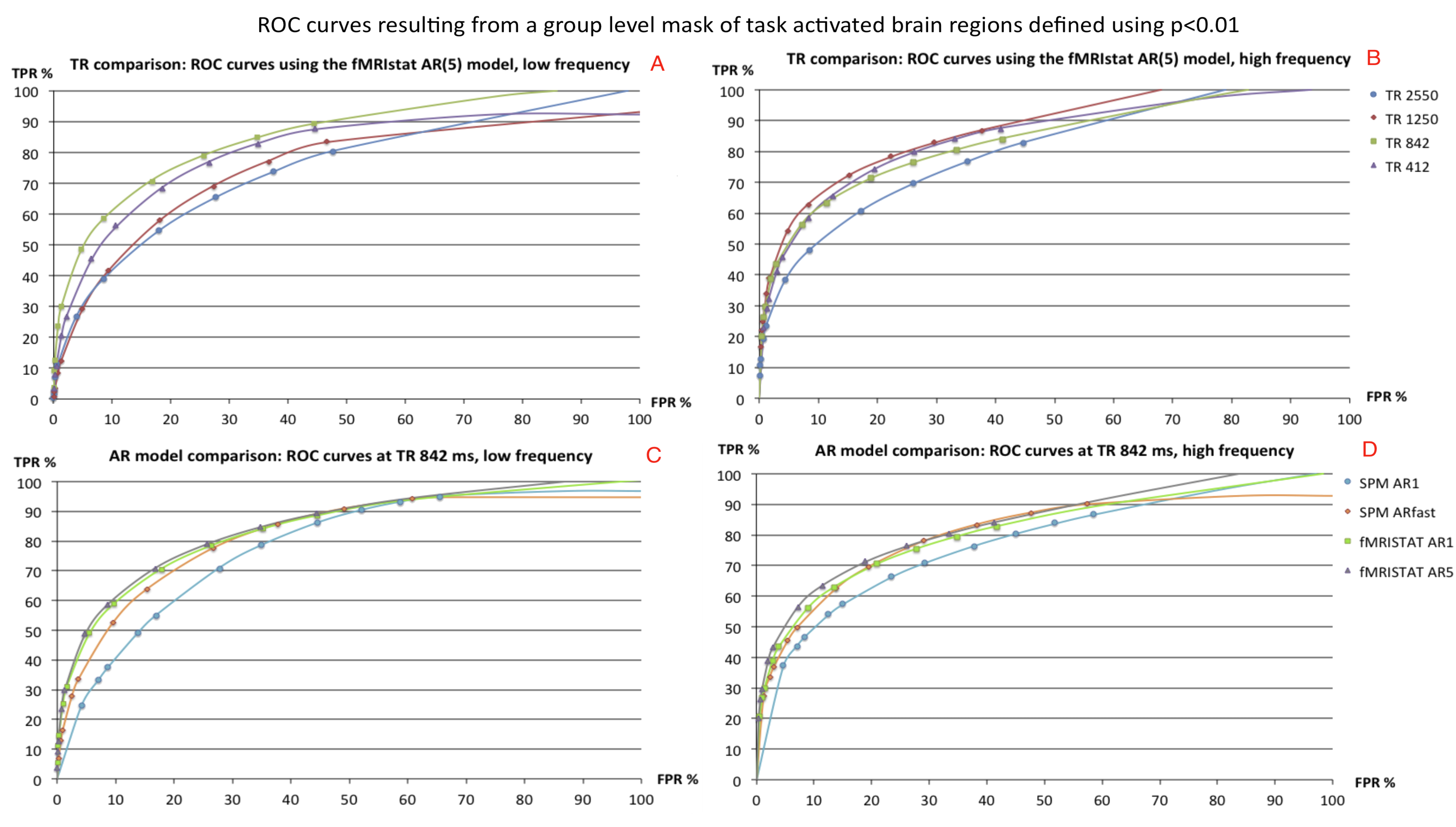


Supporting Information Figure S1: ROC curves for each TR for the lowest (A) and highest (B) rate of presentation obtained with conservative autocorrelation estimates (fMRIstat AR5). ROC curves were also calculated for each autocorrelation model at a TR of 842ms for the lowest (C) and highest rate of presentation (D). All ROC curves were produced using a group level mask (p< 0.01, 20 voxels) to determine true/false positives
